# Patterns of unplanned hospital admissions among people with dementia: from diagnosis to the end of life

**DOI:** 10.1093/ageing/afac098

**Published:** 2022-05-17

**Authors:** Emel Yorganci, Robert Stewart, Elizabeth L Sampson, Katherine E Sleeman

**Affiliations:** Cicely Saunders Institute of Palliative Care, Policy & Rehabilitation, King’s College London, Florence Nightingale Faculty of Nursing, Midwifery & Palliative Care, London, UK; Department of Psychological Medicine, Institute of Psychiatry, Psychology and Neuroscience, King's College London, London, UK; Mental Health of Older Adults, South London and Maudsley NHS Foundation Trust, London, UK; Department of Psychological Medicine, Royal London Hospital, East London NHS Foundation Trust, London, UK; Division of Psychiatry, University College London, London, UK; Cicely Saunders Institute of Palliative Care, Policy & Rehabilitation, King’s College London, Florence Nightingale Faculty of Nursing, Midwifery & Palliative Care, London, UK

**Keywords:** hospitalisation, dementia, incidence, retrospective studies, older people

## Abstract

**Background:**

hospitalisations are sentinel events for people with dementia. How patterns of unplanned hospital admissions change among people with dementia after diagnosis is relatively unknown.

**Objective:**

to describe patterns of unplanned hospital admissions of people with dementia from diagnosis until death/study end.

**Methods:**

retrospective cohort study using mental healthcare provider data of people diagnosed with dementia in London, UK (1995–2017), linked to mortality and hospital data. The primary outcome was the rate of unplanned hospital admissions after diagnosis until death/study end. We calculated the cumulative incidence of unplanned hospital admissions. The rates of unplanned hospital admissions and the percentage of time spent as an inpatient were stratified by time from first dementia diagnosis.

**Results:**

for 19,221 people with dementia (61.4% female, mean age at diagnosis 81.0 years (standard deviation, SD 8.5)), the cumulative incidence of unplanned hospital admissions (*n* = 14,759) was 76.8% (95% CI 76.3%–77.3%). Individuals remained in the study for mean 3.0 (SD 2.6) years, and 12,667 (65.9%) died. Rates and lengths of unplanned hospital admissions remained relatively low and short in the months after the dementia diagnosis, increasing only as people approached the end of life. Percentage of time spent as an inpatient was <3% for people who were alive at the study end but was on average 19.6 and 13.3% for the decedents in the last 6 and 12 months of life, respectively.

**Conclusions:**

the steep rise in hospitalisations before death highlights the need for improved community care and services for people with dementia who are approaching the end of life.

## Key Points

- Rates of unplanned hospital admissions remain relatively low and stable after dementia diagnosis and only increase as people with dementia approach the end of life.
- People who died with dementia spent 19.6 and 13.3% of their time in hospital due to unplanned hospital admissions in the last 6 and 12 months of their lives, respectively
- Investing in resources for dementia end-of-life care may help with reducing the negative impact of unplanned hospital admissions.

## Introduction

The number of people living with dementia is increasing [1–3]. As dementia and comorbidities progress, provision of care can become challenging [4, 5]. Unplanned hospital admissions (those that occur unexpectedly and urgently [6]) for people with dementia can be associated with functional and cognitive decline, though the causal relationship remains unclear [7]. Understanding when unplanned hospital admissions are most likely to occur can guide investment in the resources needed for providing high-quality dementia care.

Most studies examining unplanned hospital admissions of people with dementia have focused on specific subgroups of people with dementia (e.g. people diagnosed with Alzheimer’s disease or people who live in the community [8, 9]) or timeframes (e.g. year after diagnosis or last year of life; [10–12]). Prospective studies have been small [4, 13] or have obtained hospitalisation information from carers, and are therefore subject to recall bias. Although some people live many years following a dementia diagnosis, for others the time between diagnosis and death is shorter. Patterns of unplanned hospital admissions, and how these change before death, are relatively unknown. In this study, we aimed to describe the patterns of unplanned hospital admissions of people with dementia from the point of diagnosis.

## Methods

### Setting & data sources

This was a retrospective study using linkage between two clinical datasets (South London and Maudsley National Health Service (NHS) Foundation Trust Biomedical Research Centre’s (SLaM BRC) Clinical Record Interactive Search (CRIS) and Hospital Episode Statistics (HES)), and a death registry (Office of National Statistics—ONS). HES is a database containing details of all admissions at NHS England hospitals [14]. Electronic health records were implemented across SLaM from 2006 [15]. The CRIS application provides research access to repository of anonymised structured and open-text data from electronic health records within SLaM.

SLaM provides mental healthcare services, including dementia assessment and management to 1.2 million residents in four London boroughs (Croydon, Lambeth, Lewisham and Southwark) in the UK. Potential cases of dementia are ascertained in primary care (which has high specificity [16]), followed by referral to a specialist dementia diagnostic service (such as SLaM; [17]).

### Cohort description

The cohort was derived using the CRIS platform. All dementia diagnoses recorded in CRIS were determined from structured fields of ICD-10 diagnosis codes (F00x–03x) or supplemented by dementia diagnosis recorded in text fields by a validated natural language processing algorithm using General Architecture for Text Engineering (GATE) software [15, 18, 19]. We included any person with dementia who was 50 or older at first recorded diagnosis before 31st March 2017. HES data were available up to 31st March 2018 to allow at least 1 year of potential hospital admissions data follow-up for those who were diagnosed recently. ONS mortality records were used to retrieve information on the date of death of the decedents.

### Demographics & clinical variables

We extracted data closest to the first dementia diagnosis from CRIS on age, gender and ethnic group. Neighbourhood-level socioeconomic status was estimated using the Index of Multiple Deprivation (IMD; [20]). This is the official measure of relative deprivation in England, which encompasses living conditions of individuals from 32,844 neighbourhoods termed Lower Layer Super Output Areas (LSOAs). Each LSOA contains ~1,500 people. IMD was derived from the LSOA associated with the patient’s address (recorded closest to the diagnosis) and converted into quintiles of the national distribution (1—most deprived and 5—least deprived). We extracted dementia sub-type (Alzheimer’s disease, vascular dementia, Lewy body dementia, other or unspecified dementia (where aetiology was unrecorded)) and Mini Mental State Examination (MMSE; [21]) scores (from structured and unstructured fields) to estimate dementia severity closest to dementia diagnosis, categorising into ‘mild’ (MMSE ≥ 20), ‘moderate’ (MMSE = 10–19) or ‘severe’ (MMSE < 10).

### Outcomes

We retrieved information on non-elective hospitalisations (referred to as ‘unplanned hospital admission’) for each participant, which started after the date of their first CRIS-recorded dementia diagnosis. The primary outcome was the number of unplanned hospital admissions from the first dementia diagnosis until death or study end (31st March 2018). Unplanned hospital admissions were identified through the HES inpatient codes for a spell’s start date and admission method. Hospitalisations were defined from HES episodes, combining contiguous episodes (i.e. where start and end dates were on the same day). Numbers of unplanned hospital admissions were calculated for every 6 months from diagnosis to death or study end (31st March 2018). Six-monthly periods are commonly used for measuring care quality for people with dementia, and enable exploration of changes at a more granular level than yearly rates [22]. The secondary outcome was the percentage of time spent as an inpatient in hospital due to unplanned hospital admissions.

### Statistical analysis

We described the cohort’s sociodemographic and clinical characteristics using mean (standard deviation, SD), median (range (25th–75th percentiles)) and percentages. We calculated the cumulative incidence of unplanned hospital admissions (*= number of people admitted at least once during the study period/total number in the cohort*), and the unplanned hospital admission rate (*= all admissions per person-months were calculated as time between CRIS dementia diagnosis and death or end of the follow-up period*)) with 95% confidence intervals (CIs) and standard errors (SEs; [23]). For time spent as an inpatient, we calculated the percentage of days spent in hospital due to unplanned hospital admissions out of the total days contributed by each person. We also calculated the percentage of days spent in hospital due to unplanned hospital admissions out of the total days contributed by each person for the last 6 and 12 months of life for the decedents. We analysed the admission rates and the time spent as an inpatient for 6-monthly intervals from the point of diagnosis onwards, stratified by time to death (in years) for decedents, and time to study end date for people who remained alive.

### Ethics statement

The source database is approved for secondary analysis by the Oxfordshire Research Ethics Committee C (reference 18/SC/0372).

### Public and patient involvement

This project is part of the EMBED-Care research programme, which was developed and designed with people with dementia and their family carers from the funding application through to interpretation of these study findings [24].

## Results

### Cohort description

We obtained data on 19,221 people aged ≥50 years who had a dementia diagnosis between 1995 and 2017 (Table 1). Mean age at diagnosis was 81.0 (SD 8.5) years. Most of the cohort were women (61.4%), white (73.1%) and nearly half of the first ever recorded dementia diagnoses were Alzheimer’s disease (48.4%). In terms of the MMSE recorded closest to the diagnosis date, 40.6% of the cohort were categorised as mild (MMSE ≥20). The number of people in the cohort increased over the study period and 66.1% of people were diagnosed after 2009. Around two-thirds (65.9% (*n* = 12,667)) of the cohort died before the study end, whereas 34.1% (*n* = 6,554) of the cohort were living with dementia at the study end. Sociodemographic and clinical characteristics of the cohort stratified by time to death/study end are presented in Appendix 1 (Supplementary data are available in *Age and Ageing* online).

**Table 1:** Characteristics of people diagnosed with dementia

Characteristic	All (*n* = 19,221)	Alive at the study end date (*n* = 6,554)	Decedents (*n* = 12,667)
	%	%	%
Age at diagnosis (mean, SD)	81.0 (8.5)	78.0 (8.9)	82.6 (7.8)
Age at diagnosis categories			
50–59	2.1	3.9	1.1
60–64	2.4	4.4	1.4
65–69	4.9	8.1	3.3
70–74	10.5	13.4	8.9
75–79	18.6	22.5	16.5
80–84	24.5	23.0	25.1
85–89	23.0	17.5	26.0
90–94	10.9	6.1	13.7
≥ 95	3.1	1.2	4.3
Age at the end of the follow-up period/death (mean, SD)	84.5(8.1)	82.6 (8.6)	85.6 (7.6)
Sex			
Female	61.4	63.0	61.0
Male	38.4	37.0	39.0
Ethnicity			
White British	62.4	51.0	68.2
African/Caribbean	14.6	22.5	10.6
White other	10.7	11.8	10.2
Asian	4.3	6.5	3.2
Any other ethnicity	2.7	4.5	1.7
Mixed	0.7	1.2	0.4
Missing	4.6	2.5	5.7
IMD quintile at diagnosis			
1 (most deprived)	29.6	30.3	29.4
2	34.8	34.3	35.0
3	18.1	19.1	17.6
4	9.1	8.5	9.3
5 (least deprived)	7.2	7.1	7.3
Missing	1.2	0.7	1.4
First recorded dementia diagnosis			
Alzheimer’s disease	48.4	54.9	45.1
Vascular dementia	24.5	19.2	27.3
Unspecified dementia	23.4	22.0	24.2
Other dementia	2.7	2.8	2.6
Lewy body dementia	1.0	1.1	0.9
MMSE closest to diagnosis date			
Mild (≥20)	40.6	51.7	34.8
Moderate (10–19)	32.82	29.6	24.5
Severe (<10)	7.4	5.5	8.4
Missing	19.2	13.2	32.3

IMD, index of multiple deprivation (1 = most deprived, 5 = most affluent); MMSE, mini-mental state examination; SD, standard deviation.

In total, there were 54,017 unplanned hospital admissions. Cumulative incidence rate was 76.8% (95% CI 76.3–77.3%); 14,759 people had at least one unplanned hospital admission. Of all unplanned hospital admissions, 20,140 (37.3%) took place in people who were in the last year of life. The median number of unplanned hospital admissions for the whole cohort was 3(1–5). The median time spent as an inpatient per admission was 5 (1–14) days per person. The mean total time spent in the hospital for the whole cohort was 32 (11–67) days. The median time spent as an inpatient in one unplanned hospital admission in the last year of life was 7 (2–18) days per person. The total time spent in the hospital in the last year of life was 30 (12–59) days per person.

### The decedents

The mean age at diagnosis was 82.6 (SD 7.8); mean age at death 85.6 (SD 7.6). Decedents accounted for 72.7% (*n* = 10,735/14,759) of all people who had an unplanned hospital admission. The cumulative incidence rate of unplanned hospital admissions for the decedents was 84.8% (95% CI 84.1–85.4%). Over a third of decedents (38.1%, *n* = 4,697) died in hospital and 24.5% of these people (*n* = 1,153) died during their first admission after diagnosis.

**Figure 1:**
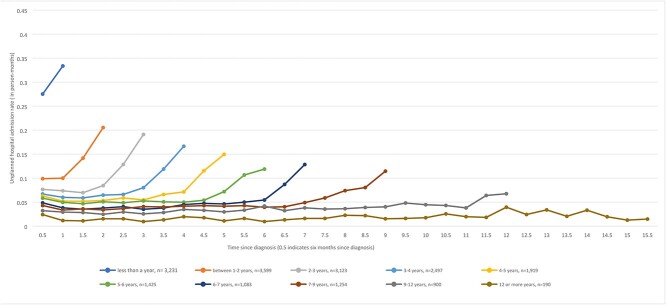
Unplanned hospital admission rates for people who died with dementia (*n* = 12,667) by duration of survival after diagnosis.

Admission rates for decedents ranged from 3 to 334 per 1,000 person-months (Figure 1). In the first 6 months after diagnosis, higher admission rates were observed in the subgroup of people who lived less than a year after diagnosis. For subgroups of people who lived longer than 2 years after diagnosis, the following pattern was observed: admission rates in the first 6 months were low and remained relatively stable until the last 12 months of life when they increased steeply. Patterns of rates of unplanned hospital admissions for the whole cohort, and details of admission rates (95% CIs and SEs) are provided in Appendix 2 (Supplementary data are available in *Age and Ageing* online).

**Figure 2:**
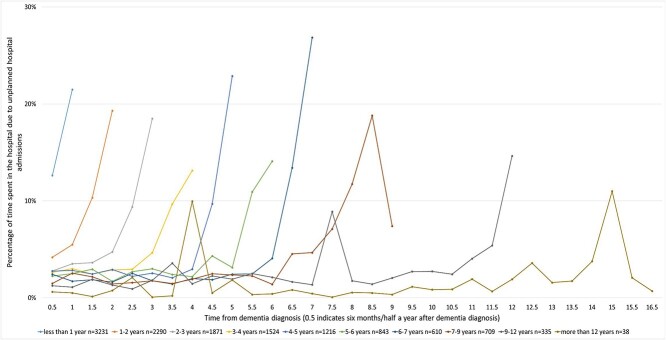
Percentage of time (6-month periods) spent in the hospital due to unplanned hospital admissions for people who died with dementia (*n* = 12,667) by duration of survival after diagnosis.

Similarly, the percentage of days spent as an inpatient in hospital increased in the last year of life (Figure 2). Across the subgroups, percentage of days spent as an inpatient due to unplanned hospital admissions ranged between 0.6 and 12.6% in the first 6 months after diagnosis, and was highest for those with shortest survival. For people who lived with dementia for more than a year, percentage of days spent in hospital was low after diagnosis and increased towards the end of life. The decedents spent on average 19.6% (SD 3.1) and 13.3% (SD 2.5) of their time in hospital due to unplanned hospital admissions in the last 6 months and the last year of their lives, respectively.

### People who were living at the study end

Mean age at diagnosis varied between 83.7 (SD7.7) and 72.5 (SD 9.9) across the subgroups (Appendix 2, Supplementary data are available in *Age and Ageing* online). People who were living with dementia at study end accounted for 27.3% (*n* = 4,024) of 19,211 people. The cumulative incidence rate of unplanned hospital admissions for people who were living with dementia at the study end was 61.4% (95% CI 60.2–62.6%).

**Figure 3:**
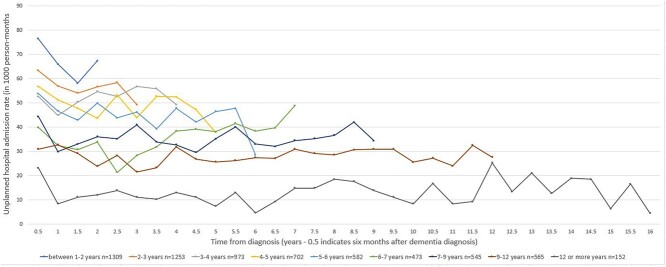
Unplanned hospital admission rates for surviving people with dementia (*n* = 6,554) by duration of time after diagnosis.

**Figure 4:**
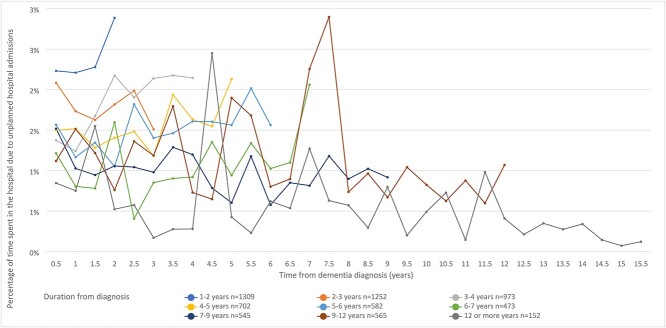
Percentage of time (6-month periods) spent in the hospital due to unplanned hospital admissions for surviving people with dementia (*n* = 6,554) by duration of time after diagnosis.

Admission rates for people who were living with dementia at the study end ranged from 4 to 77 per 1,000 person-months (Figure 3). Across the subgroups, a drop in the admission rates in the first 6-monthly interval after diagnosis was observed. The unplanned hospital admission rates remained relatively low and stable over the years after dementia diagnosis. Higher admission rates were observed for people who were diagnosed more recently compared to those who had been living with a dementia diagnosis for a longer time. Considering the total time after diagnosis, the percentage of time spent as inpatient in hospital due to unplanned hospital admissions for people were living with dementia at the study end ranged from 0.08 to 2.9% (Figure 4). Compared to the decedents, percentage time spent as inpatient in hospital due to unplanned hospital admissions remained low and stable across the subgroups.

## Discussion

In this study, over three quarters (76.8%) of a large sample of people with dementia experienced at least one unplanned hospital admission after diagnosis. Rates of unplanned hospital admissions remained relatively low and stable after dementia diagnosis and did not increase for people who were living with dementia at the study end. Rates and lengths of unplanned hospital admissions increased steeply as people approached the end of their lives, regardless of survival duration. Higher rates of unplanned hospital admissions were observed for people who were diagnosed for less than a year. Admissions in the last year of life accounted for 37.3% of all unplanned hospital admissions for the whole cohort.

The cumulative incidence rate of unplanned hospital admissions reported in our study is similar to the one (75.9%) reported by a study of people with dementia from the same mental health trust for a shorter time interval (2008–2016) [12]. Previously reported cumulative incidence of hospitalisations of people with dementia with varying follow-up times ranged between 23.6 and 86.0% [8, 25, 26]. Lower hospitalisation rates have been reported in studies with people who were not approaching the end of their lives. This is also evident in our study, where the cumulative incidence rate was lower for people who were living at the end of follow-up (61.4%) compared to the decedents (84.8%). A similar cumulative incidence rate of 80.8% was observed in UK for people older than 75 who had at least one unplanned hospital admission in the last year of life [27].

Comparisons of the hospitalisation rates of people with dementia and those other life-limiting conditions have shown mixed results [8, 28, 29]. Rates of unplanned hospital admissions of people with other life-limiting illnesses such as cancer and organ failure also increase towards death [30, 31]. An overlap between dementia and other factors (e.g. physical, psychological, financial, carer strain, service availability and dementia care expertise) is likely to drive hospitalisations towards the end of life [10]. For people with dementia, hospital environments may be distressing. If appropriate, dementia care in the community may be a better option towards the end of life [4]. Most people with dementia would prefer to live and die in a care home or at home [32]. However, in practice, it is difficult to determine when a person with dementia is approaching death [33]. In our sample, 38.1% died in the hospital, which is similar to national data [34] and 9.1% died on their first unplanned hospital admission after diagnosis. Most dementia deaths occur in care homes, therefore a smaller percentage of people with dementia die in hospitals compared to the general population [35, 36].

Access to palliative care, living in care homes and having continuity of care (e.g. consulting the same general practitioner (GP) consistently) are associated with reduced hospital admissions among people with dementia who may be approaching death [37–40]. Yet, people with dementia experience inequitable access to high-quality palliative and end-of-life care [41]. In recent years, decline in the number of care home beds and in continuity in general practices have been observed in the UK [42, 43]. If primary and community care services are not equipped to address complex needs of people with dementia who are approaching the end of life, admission rates are likely to remain high. Lack of support for informal carers may also lead to waiting until a crisis point, which may then require longer hospital stays [44]. Discussing and setting ceilings of care with people with dementia and carers regularly, and investing in community care and care homes may make meeting care preferences more likely [32]. However, interventions for avoiding hospitalisations for people with dementia have had disappointing results [45]. Future research should focus on reducing the length of time spent as inpatient, whereas improving dementia end-of-life care in hospitals. Unplanned hospital admissions and length of time spent in the hospital in the last year of life can be used as indicators of how well the health and social care systems are working for people with dementia and their families [46].

A strength of our study was the large sample, which was not limited to decedents and included people living at home and in care homes, with any dementia diagnosis and severity, thus reducing biases, which may be introduced by subject and time period selection [47]. Recruiting and retaining people with dementia in research studies is challenging [48]. Using routinely collected data permitted observation of trends for over 19,000 people of dementia of whom lived with a diagnosis for varying durations. Although not best practice, people may be diagnosed with dementia during a hospital admission; in our analysis we included only unplanned hospital admissions, which started after the dementia diagnosis date, as we were interested in hospital admission among people with an existing diagnosis of dementia. The identification of dementia diagnosis was limited to records of a single mental health trust, which may under-record dementia diagnoses and limit generalisability [17, 49]. People may have been diagnosed at an earlier date than that recorded in CRIS [17]. The proportion of people with a dementia diagnosis is relatively high in this catchment area compared to national figures [12, 50]. However, the average age at diagnosis and death, and the duration between diagnosis and death from our findings are consistent with national averages [51]. Finally, this was a descriptive study using routine data; we did not test for differences or predictors of unplanned hospital admissions, which have been examined previously [26]. By leveraging the linkage between a mental health trust and national hospital data, we were able to have a near-complete picture of unplanned hospital admissions. Only 1% of UK hospital services are not provided by the NHS and these are likely to be less relevant for unplanned hospital admissions of people with dementia [52].

Although the number of people affected by dementia is increasing, understanding of the disease development and progression remains low among the public and healthcare professionals [53, 54]. Most hospitals provide training around caring for people with dementia but many do not include specific skills needed for care of those approaching the end of life [55]. Lack of confidence by staff and negative attitudes towards dementia in hospitals may lead to poor quality of care [56]. Opportunities to improve public perception of dementia as a neurodegenerative, terminal illness [54] and provision of dementia and end-of-life care training for healthcare professionals should be further developed where necessary and adopted by health policy.

## Conclusion

A steep increase in the rates and lengths of unplanned hospital admissions occurs among people with dementia as they approach the end of life. This may indicate insufficient community resources for meeting care needs. Many people with dementia, and their loved ones, would prefer to spend less time in hospital towards the end of life [57]. Reducing the burden of unplanned hospital admissions will require well-resourced, high-quality, dementia and end-of-life care both in hospitals and community settings. Efforts to avoid unnecessary hospital admissions, long admission durations and readmissions must be prioritised.

## Supplementary Material

aa-21-1965-File001_afac098
