# Supplementary material for: Patterns of unplanned hospital admissions among people with dementia: from diagnosis to the end of life
Source: Age Ageing. 2022 May 17;51(5):afac098. doi: 10.1093/ageing/afac098 (PMC9113942; doi:10.1093/ageing/afac098)
Supplement: aa-21-1965-File001_afac098 [file aa-21-1965-file001_afac098.docx]

Manuscript Title: Patterns of unplanned hospital admissions among people with dementia: From diagnosis to the end of life

# Appendix I

Table 1. Characteristics of people with dementia stratified by time to death/ end of the follow-up period (n=19,221)

|  | **Decedents (n=12,667)** | | | | | | | | | | **People who were alive at the study end (n=6,554)** | | | | | | | | |
| --- | --- | --- | --- | --- | --- | --- | --- | --- | --- | --- | --- | --- | --- | --- | --- | --- | --- | --- | --- |
| **Follow-up since dementia diagnosis**  **(years)** | <1 (n=3,231) | 1 – 2 (n=2,290) | 2 – 3 (n=1,871) | 3 – 4 (n=1,524) | 4 – 5  (n=1,216) | 5 – 6 (n=843) | 6 – 7  (n=610) | 7 – 9 (n=709) | 9 – 12 (n=335) | >12 (n=38) | 1 – 2 (n=1,309) | 2 – 3 (n=1,252) | 3 – 4 (n=973) | 4 – 5  (n=703) | 5 – 6 (n=582) | 6 – 7  (n=473) | 7 – 9 (n=545) | 9 – 12 (n=565) | >12 (n=152) |
| **Had at least one unplanned hospital admission after dementia diagnosis (%)**  **(n=14,759)** | 66.8 | 87.0 | 89.9 | 92.3 | 92.4 | 93.1 | 93.1 | 94.2 | 94.3 | 97.4 | 46.6 | 56.9 | 85.1 | 69.1 | 71.0 | 69.8 | 69.5 | 68.5 | 65.8 |
| **Gender (%)**  Female | 57.1 | 57.3 | 61.6 | 60.8 | 64.9 | 63.6 | 66.7 | 70.1 | 69 | 60.5 | 64.9 | 65.5 | 65.7 | 61.8 | 35.1 | 34.5 | 34.3 | 34.3 | 38.2 |
| **Ethnicity (%)**  Black  Asian  Other ethnic group  White  Missing | 8  2.9  2.4  76.8  9.9 | 9.4  3.6  2.8  78.4  5.9 | 11.4  2.8  2.4  79.3  5.9 | 9.9  2.5  1.3  82.7  3.6 | 11.9  3.2  1.6  79.7  3.5 | 12.5  4.4  1.6  75.4  4.3 | 14.3  4.4  1.6  75.4  4.3 | 13.7  3.8  1.8  76.7  4.0 | 17.3  3.6  1.5  73.1  4.5 | 18.4  2.6  2.6  73.7  2.6 | 23.5  6.0  3.4  62.6  4.4 | 21.8  6.8  9.0  59.7  2.7 | 23.1  6.8  9.0  59.7  2.7 | 20.9  4.8  3.1  68.9  2.3 | 21.0  6.7  3.1  67.7  1.6 | 23.0  7.2  3.8  64.7  1.3 | 23.9  7.3  4.4  63.5  0.9 | 23.7  5.8  4.8  63.9  1.8 | 17.8  10.5  5.9  58.6  7.2 |
| **Age at diagnosis**  **Mean (SD)** | 84.7 (7.3) | 83.7 (7.7) | 83.1(7.4) | 82 (7.7) | 81.4(7.5) | 80.4 (7.6) | 79.5  (7.9) | 78.6 (7.6) | 76.8  (7.9) | 74.1  (9.2) | 80.3  (8.6) | 79.7  (8.0) | 78.9  (8.6) | 77.8  (9.1) | 77.8 (8.2) | 76  (8.8) | 74.5  (9.1) | 73.7  (8.8) | 72.5  (9.9) |
| **Age at death/window end Mean (SD)** | 85.1 (7.1) | 85.2 (7.7) | 85.6 (7.4) | 85.5 (7.7) | 85.9 (7.5) | 85.8 (7.6) | 85.9 (8.0) | 86.5 (7.6) | 86.9 (7.9) | 87.4 (9.1) | 81.8 (8.6) | 82.2 (8.0) | 82.4 (8.6) | 82.3 (9.0) | 83.3 (8.2) | 82.5 (8.8) | 82.3 (9.1) | 84.1 (8.8) | 85.9 (9.9) |
| **First recorded dementia diagnosis (%)**  Alzheimer’s disease  Vascular dementia  Unspecified dementia  Lewy body dementia  Other dementia | 36.5  30.5  29.6  1  2.5 | 42.4  30.2  24.1  1.1  2.3 | 47.4  25.3  24.0  1.2  2.1 | 49.1  24.0  22.5  1.1  3.4 | 47.0  28.5  21.3  0.3  2.9 | 52.0  23.8  20.6  0.8  2.7 | 53.8  22.5  20.8  0.5  2.5 | 52.8  24.1  20.0  0.6  2.5 | 57.3  23.3  16.7  0  2.7 | 63.2  23.7  7.9  0  5.3 | 52.9  17.3  26.0  1.8  2.1 | 59.4  16.9  20.9  0.9  2.0 | 60.6  17.7  17.6  1.3  2.8 | 55.3  19.2  20.8  1.0  3.7 | 55.3  19.1  22.9  1.2  1.6 | 52.9  18.0  26.0  0.2  3.0 | 53.2  22.8  19.8  1.1  3.1 | 45.1  25.3  23.2  0.5  5.8 | 44.7  31.6  17.8  0.7  5.3 |
| **MMSE^a^ nearest to the diagnosis (%)**  **(n=12, 236)** | 17.1(6.4) | 17.6 (6.4) | 17.6 (6.4) | 18.2 (6.4) | 18.6 (6.2) | 18.7 (6.2) | 18.8 (6.2) | 19.1 (6.3) | 18.4 (6.7) | 20.6 (6.9) | 20.2 (6.2) | 20.0 (6.1) | 19.4 (6.1) | 20.1 (6.0) | 20.6 (5.8) | 20.2 (6.3) | 20.4 (6.1) | 20.0 (6.2) | 20.8 (6.8) |
| **MMSE categories (%)**  Mild (≥20)  Moderate (10-19)  Severe (<10)  Missing | 24.7  32.7  8.4  34.2 | 31.8  35.6  9.2  23.4 | 34.4  37.2  9.8  18.6 | 40.7  33.7  8.7  15.3 | 40.6  37.1  7.0  15.3 | 42.6  36.9  6.8  13.8 | 43.6  33.0  8.0  15.4 | 46.7  30.8  6.2  16.4 | 43.0  30.5  9.6  17.0 | 57.9  18.4  7.9  15.8 | 49.6  30.6  4.8  15.0 | 50.6  29.7  7.0  8.8 | 50.8  33.4  7.0  8.8 | 53.1  29.0  5.6  12.4 | 57.7  27.8  4.6  9.8 | 55.2  29.2  6.6  9.1 | 55.8  27.7  5.3  11.2 | 49.0  28.0  5.3  17.7 | 40.1  17.8  4.6  37.5 |
| **IMD Quintiles (%)** |  |  |  |  |  |  |  |  |  |  |  |  |  |  |  |  |  |  |  |
| 1 (most deprived)  2  3  4  5 (least deprived)  Missing | 29.9  35.8  16.6  9.1  6.9  1.7 | 30.1  33.7  18.2  10.1  6.9  1.1 | 28.8  35.0  17.4  9.6  7.6  1.7 | 29.3  34.1  18.2  9.3  7.6  1.6 | 29.5  34.1  18.8  9.4  6.8  1.4 | 27.5  34.2  19.3  8.5  8.8  1.7 | 27.9  37.4  14.9  9.7  8.4  1.8 | 29.3  35.4  18.2  9.3  6.5  1.3 | 26.6  40.0  16.7  6.0  9.3  1.5 | 18.4  42.1  15.8  10.5  13.2  0 | 30.9  35.8  18.2  8.1  6.3  0.8 | 29.4  35.9  18.8  8.1  7.2  0.7 | 32.0  31.8  19.1  9.0  7.4  0.7 | 26.3  37.4  19.5  9.0  7.5  0.3 | 26.5  32.5  21.8  9.3  9.5  0.5 | 31.5  34.5  19.5  7.4  7.0  0.2 | 31.6  33.0  18.2  10.3  6.6  0.4 | 34.0  30.3  20.0  8.0  6.4  1.4 | 31.6  37.5  14.5  7.9  4.6  4.0 |

^a^MMSE scores range between

# Appendix II

Table 1. Incidence rates and 95% confidence intervals (CIs) for unplanned hospital admissions of people with dementia (n=19,221) – Corresponding to Figure 1, Appendix 2

| *Six- monthly time intervals from time of diagnosis (0) to death* | *0.5* | *1* | *1.5* | *2* | *2.5* | *3* | *3.5* | *4* | *4.5* | *5* | *5.5* | *6* | *6.5* | *7* |
| --- | --- | --- | --- | --- | --- | --- | --- | --- | --- | --- | --- | --- | --- | --- |
| *Duration between diagnosis and death (number of people) (number of admissions)* | *Rate  95% CIs - Lower limit  95% CIs - Upper Limit Standard Error* | | | | | | | | | | | | | |
|  |  |  |  |  |  |  |  |  |  |  |  |  |  |  |
| less than one-year n=3,231 | 275.2 | 333.7 |  |  |  |  |  |  |  |  |  |  |  |  |
| n-4,226 | 265.5 | 315.4 |  |  |  |  |  |  |  |  |  |  |  |  |
|  | 285.2 | 352.7 |  |  |  |  |  |  |  |  |  |  |  |  |
|  | 5 | 9.5 |  |  |  |  |  |  |  |  |  |  |  |  |
|  |  |  |  |  |  |  |  |  |  |  |  |  |  |  |
| between 1-2 years n=3,599 | 98.8 | 99.9 | 141.6 | 205.3 |  |  |  |  |  |  |  |  |  |  |
| n=7,538 | 94.6 | 95.7 | 135.8 | 193.4 |  |  |  |  |  |  |  |  |  |  |
|  | 103 | 104.2 | 147.5 | 217.8 |  |  |  |  |  |  |  |  |  |  |
|  | 2.1 | 2.2 | 3 | 6.2 |  |  |  |  |  |  |  |  |  |  |
|  |  |  |  |  |  |  |  |  |  |  |  |  |  |  |
| 2-3 years n=3,123 | 76.5 | 73.6 | 69.6 | 84.4 | 128.6 | 191 |  |  |  |  |  |  |  |  |
| n=8,367 | 72.6 | 69.8 | 65.9 | 80.3 | 122.7 | 178.6 |  |  |  |  |  |  |  |  |
|  | 80.5 | 77.5 | 73.4 | 88.6 | 134.6 | 204.1 |  |  |  |  |  |  |  |  |
|  | 2 | 2 | 1.9 | 2.1 | 3 | 6.5 |  |  |  |  |  |  |  |  |
|  |  |  |  |  |  |  |  |  |  |  |  |  |  |  |
| 3-4 years n=2,479 | 66.8 | 59.9 | 58.7 | 64.6 | 65.9 | 80 | 118.9 | 166.3 |  |  |  |  |  |  |
| n=7,865 | 62.8 | 56.1 | 55 | 60.6 | 61.9 | 75.6 | 112.6 | 153.7 |  |  |  |  |  |  |
|  | 71 | 64 | 62.7 | 68.8 | 70.2 | 84.7 | 125.5 | 179.7 |  |  |  |  |  |  |
|  | 2.1 | 2 | 2 | 2.1 | 2.1 | 2.3 | 3.3 | 6.6 |  |  |  |  |  |  |
|  |  |  |  |  |  |  |  |  |  |  |  |  |  |  |
| 4-5 years n=1,919 | 62.7 | 52 | 51.7 | 53.3 | 58.7 | 54.6 | 65.9 | 71.3 | 115.3 | 149.5 |  |  |  |  |
| n=6,821 | 58.3 | 47.9 | 47.7 | 49.2 | 54.4 | 50.4 | 61.3 | 66.5 | 108.3 | 135.8 |  |  |  |  |
|  | 67.4 | 56.3 | 56 | 57.6 | 63.3 | 59 | 70.7 | 76.3 | 122.7 | 164.3 |  |  |  |  |
|  | 2.3 | 2.1 | 2.1 | 2.2 | 2.3 | 2.2 | 2.4 | 2.5 | 3.7 | 7.3 |  |  |  |  |
|  |  |  |  |  |  |  |  |  |  |  |  |  |  |  |
| 5-6 years n=1,425 | 58.2 | 49.5 | 46.7 | 50.8 | 48.7 | 52.5 | 50.5 | 49.8 | 53.9 | 71.8 | 106.5 | 118.8 |  |  |
| n=5,470 | 53.3 | 44.9 | 42.3 | 46.2 | 44.1 | 47.8 | 45.9 | 45.3 | 49.2 | 66.3 | 98.7 | 104.6 |  |  |
|  | 63.5 | 54.4 | 51.4 | 55.7 | 53.5 | 57.5 | 55.5 | 54.7 | 59 | 77.7 | 114.8 | 134.4 |  |  |
|  | 2.6 | 2.4 | 2.3 | 2.4 | 2.4 | 2.5 | 2.4 | 2.4 | 2.5 | 2.9 | 4.1 | 7.6 |  |  |
|  |  |  |  |  |  |  |  |  |  |  |  |  |  |  |
| 6-7 years n=1,083 | 48.5 | 38 | 35.3 | 37.6 | 40.5 | 35.1 | 37.7 | 44.9 | 47.4 | 46.2 | 49.8 | 54.4 | 86.9 | 128.3 |
| n=3,971 | 43.3 | 33.5 | 30.9 | 33.1 | 35.8 | 30.8 | 33.2 | 40 | 42.3 | 41.2 | 44.6 | 49 | 78.8 | 111.4 |
|  | 54 | 43 | 40 | 42.5 | 45.6 | 39.9 | 42.7 | 50.3 | 52.9 | 51.6 | 55.5 | 60.3 | 95.6 | 147.1 |
|  | 2.7 | 2.4 | 2.3 | 2.4 | 2.5 | 2.3 | 2.4 | 2.6 | 2.7 | 2.7 | 2.8 | 2.9 | 4.3 | 9.1 |
|  |  |  |  |  |  |  |  |  |  |  |  |  |  |  |
| 7-9 years n=1,254 | 42.9 | 33.5 | 35.9 | 33.7 | 36.5 | 40.7 | 39.9 | 41.5 | 42.7 | 41.5 | 42.7 | 39.3 | 40.3 | 48.9 |
| n=5,138 |  |  |  |  |  |  |  |  |  |  |  |  |  |  |
|  | 38.4 | 29.5 | 31.8 | 29.8 | 32.4 | 36.3 | 35.5 | 37 | 38.2 | 37 | 38.2 | 35 | 35.9 | 44.1 |
|  | 47.8 | 37.8 | 40.3 | 38.1 | 41.1 | 45.4 | 44.6 | 46.3 | 47.5 | 46.3 | 47.5 | 44 | 45 | 54.1 |
|  | 2.4 | 2.1 | 2.2 | 2.1 | 2.2 | 2.3 | 2.3 | 2.4 | 2.4 | 2.4 | 2.4 | 2.3 | 2.3 | 2.6 |
|  |  |  |  |  |  |  |  |  |  |  |  |  |  |  |
| 9-12 years n=900 | 32.2 | 29.2 | 28.3 | 24.6 | 29.4 | 25.5 | 28.3 | 34.8 | 32.9 | 29.6 | 33.3 | 41.8 | 32.2 | 38.1 |
| n=3,866 | 27.7 | 24.9 | 24.1 | 20.7 | 25.1 | 21.5 | 24.1 | 30.1 | 28.4 | 25.3 | 28.7 | 36.6 | 27.7 | 33.2 |
|  | 37.2 | 34.1 | 33.1 | 29.1 | 34.3 | 30.1 | 33.1 | 40 | 38 | 34.5 | 38.4 | 47.5 | 37.2 | 43.6 |
|  | 2.4 | 2.3 | 2.3 | 2.1 | 2.3 | 2.2 | 2.3 | 2.5 | 2.5 | 2.3 | 2.5 | 2.8 | 2.4 | 2.7 |
|  |  |  |  |  |  |  |  |  |  |  |  |  |  |  |
| more than 12 years n=190 | 24 | 11.6 | 10.9 | 15.3 | 15.3 | 9.5 | 13.1 | 19.7 | 17.5 | 10.9 | 16 | 9.5 | 13.1 | 16 |
| n=665 | 16.8 | 6.9 | 6.4 | 9.7 | 9.7 | 5.3 | 8 | 13.2 | 11.5 | 6.4 | 10.3 | 5.3 | 8 | 10.3 |
|  | 33.3 | 18.5 | 17.6 | 22.9 | 22.9 | 15.7 | 20.3 | 28.2 | 25.6 | 17.6 | 23.8 | 15.7 | 20.3 | 23.8 |
|  | 4.2 | 2.9 | 2.8 | 3.3 | 3.3 | 2.6 | 3.1 | 3.8 | 3.6 | 2.8 | 3.4 | 2.6 | 3.1 | 3.4 |
|  |  |  |  |  |  |  |  |  |  |  |  |  |  |  |
| *Six- monthly time intervals from time of diagnosis (0) to death* | *7.5* | *8* | *8.5* | *9* | *9.5* | *10* | *10.5* | *11* | *11.5* | *12* | *12.5* | *13* | *13.5* | *14* |
| 7-9 years n=1,254 | 58.7 | 73.9 | 80.3 | 114.5 |  |  |  |  |  |  |  |  |  |  |
| n=5,138 |  | 0 |  |  |  |  |  |  |  |  |  |  |  |  |
|  | 53.3 | 66 | 69.3 | 91.1 |  |  |  |  |  |  |  |  |  |  |
|  | 64.5 | 82.5 | 92.7 | 142 |  |  |  |  |  |  |  |  |  |  |
|  | 2.9 | 4.2 | 6 | 13 |  |  |  |  |  |  |  |  |  |  |
|  |  |  |  |  |  |  |  |  |  |  |  |  |  |  |
| 9-12 years n=900 | 35.5 | 36.3 | 38.8 | 40.1 | 48.1 | 44.5 | 43 | 37.9 | 63.9 | 67.4 |  |  |  |  |
| n=3,866 | 30.8 | 31.4 | 33.9 | 35.1 | 42.2 | 38.2 | 35.8 | 30.1 | 49.7 | 39.4 |  |  |  |  |
|  | 40.8 | 41.6 | 44.4 | 45.8 | 54.6 | 51.7 | 51.3 | 47.3 | 80.8 | 108.4 |  |  |  |  |
|  | 2.6 | 2.6 | 2.7 | 2.7 | 3.2 | 3.4 | 4 | 4.4 | 7.9 | 17.4 |  |  |  |  |
|  |  |  |  |  |  |  |  |  |  |  |  |  |  |  |
| more than 12 years n=190 | 16 | 22.6 | 21.8 | 15.3 | 16 | 17.5 | 25.5 | 19.7 | 18.2 | 39.4 | 24.2 | 33.8 | 20.4 | 33.1 |
| n=665 | 10.3 | 15.6 | 15 | 9.7 | 10.3 | 11.5 | 18 | 13.2 | 12.1 | 27.9 | 16.7 | 23.6 | 11.7 | 20.3 |
|  | 23.8 | 31.6 | 30.7 | 22.9 | 23.8 | 25.6 | 35 | 28.2 | 26.4 | 54.2 | 34.1 | 47.1 | 33.4 | 51.2 |
|  | 3.4 | 4.1 | 4 | 3.3 | 3.4 | 3.6 | 4.3 | 3.8 | 3.6 | 6.7 | 4.4 | 6 | 5.5 | 7.8 |
|  |  |  |  |  |  |  |  |  |  |  |  |  |  |  |

Table 2. Incidence rates and 95% confidence intervals (CIs) for unplanned hospital admissions of people who died with dementia (n=12,677) – Corresponding to Figure 1, Manuscript

| *Six- monthly time intervals from time of diagnosis (0) to death* | *0.5* | *1* | *1.5* | *2* | *2.5* | *3* | *3.5* | *4* | *4.5* | *5* | *5.5* | *6* | *6.5* | *7* |
| --- | --- | --- | --- | --- | --- | --- | --- | --- | --- | --- | --- | --- | --- | --- |
| *Duration between diagnosis and death (number of people) (number of admissions* | *Rate*  *95% CIs - Lower limit*  *95% CIs - Upper Limit*  *Standard Error* | | | | | | | | | | | | | |
|  |  |  |  |  |  |  |  |  |  |  |  |  |  |  |
| less than one-year n=3,231 | 275.2 | 333.7 |  |  |  |  |  |  |  |  |  |  |  |  |
| n-4,226 | 265.5 | 315.4 |  |  |  |  |  |  |  |  |  |  |  |  |
|  | 285.2 | 352.7 |  |  |  |  |  |  |  |  |  |  |  |  |
|  | 5 | 9.5 |  |  |  |  |  |  |  |  |  |  |  |  |
|  |  |  |  |  |  |  |  |  |  |  |  |  |  |  |
| between 1-2 years n=2,990 | 111.2 | 118.9 | 190 | 288.5 |  |  |  |  |  |  |  |  |  |  |
| n=6,018 | 105.7 | 113.2 | 181.6 | 270.6 |  |  |  |  |  |  |  |  |  |  |
|  | 116.9 | 124.7 | 198.7 | 307.3 |  |  |  |  |  |  |  |  |  |  |
|  | 2.8 | 2.9 | 4.4 | 9.4 |  |  |  |  |  |  |  |  |  |  |
|  |  |  |  |  |  |  |  |  |  |  |  |  |  |  |
| 2-3 years n=1,871 | 85.3 | 84.7 | 79.9 | 103 | 175.3 | 285.8 |  |  |  |  |  |  |  |  |
| n=6,211 | 80.1 | 79.5 | 74.8 | 97.2 | 166.6 | 266.1 |  |  |  |  |  |  |  |  |
|  | 90.9 | 90.2 | 85.3 | 109 | 184.4 | 306.5 |  |  |  |  |  |  |  |  |
|  | 2.8 | 2.7 | 2.7 | 3 | 4.6 | 10.3 |  |  |  |  |  |  |  |  |
|  |  |  |  |  |  |  |  |  |  |  |  |  |  |  |
| 3-4 years n=1,524 | 75.9 | 69.6 | 64.1 | 71 | 74.5 | 94.9 | 157.9 | 228 |  |  |  |  |  |  |
| n=5,747 | 60.5 | 47.3 | 48.9 | 53.4 | 69 | 88.8 | 148.6 | 209.7 |  |  |  |  |  |  |
|  | 72.3 | 57.8 | 59.5 | 64.6 | 80.2 | 101.4 | 167.5 | 247.4 |  |  |  |  |  |  |
|  | 2.9 | 2.8 | 2.6 | 2.8 | 2.9 | 3.2 | 4.8 | 9.6 |  |  |  |  |  |  |
|  |  |  |  |  |  |  |  |  |  |  |  |  |  |  |
| 4-5 years n=1,216 | 66.2 | 52.4 | 54 | 58.8 | 62 | 60.7 | 73.5 | 82.1 | 157.1 | 224.9 |  |  |  |  |
| n=4,932 | 60.5 | 47.3 | 48.9 | 53.4 | 56.4 | 55.3 | 67.4 | 75.7 | 146.7 | 203.1 |  |  |  |  |
|  | 72.3 | 57.8 | 59.5 | 64.6 | 67.9 | 66.6 | 79.9 | 88.9 | 168.1 | 248.4 |  |  |  |  |
|  | 3 | 2.7 | 2.7 | 2.8 | 2.9 | 2.9 | 3.2 | 3.4 | 5.4 | 11.6 |  |  |  |  |
|  |  |  |  |  |  |  |  |  |  |  |  |  |  |  |
| 5-6 years n=843 | 61.3 | 51.4 | 49.2 | 51.4 | 52 | 56.9 | 58.3 | 51.2 | 62.1 | 89.4 | 146.4 | 181.3 |  |  |
| n=3,722 | 54.7 | 45.4 | 43.4 | 45.4 | 46 | 50.6 | 52 | 45.3 | 55.5 | 81.4 | 134.6 | 158.5 |  |  |
|  | 68.4 | 57.9 | 55.6 | 57.9 | 58.6 | 63.8 | 65.3 | 57.7 | 69.2 | 97.9 | 159 | 206.4 |  |  |
|  | 3.5 | 3.2 | 3.1 | 3.2 | 3.2 | 3.4 | 3.4 | 3.2 | 3.5 | 4.2 | 6.2 | 12.2 |  |  |
|  |  |  |  |  |  |  |  |  |  |  |  |  |  |  |
| 6-7 years n=610 | 55.2 | 42.3 | 38.8 | 40.4 | 55.5 | 40.4 | 42.3 | 50 | 53.8 | 52.5 | 56.3 | 66.9 | 122.7 | 196.3 |
| n=2,670 | 36 | 30.8 | 32.6 | 26.9 | 48.2 | 34.3 | 36.1 | 43.1 | 46.7 | 45.4 | 49 | 58.9 | 110 | 168 |
|  | 48.3 | 42.3 | 44.3 | 37.7 | 63.5 | 47.4 | 49.4 | 57.6 | 61.7 | 60.3 | 64.4 | 75.7 | 136.5 | 228.2 |
|  | 3.9 | 3.4 | 3.3 | 3.3 | 3.9 | 3.3 | 3.4 | 3.7 | 3.8 | 3.8 | 3.9 | 4.3 | 6.8 | 15.3 |
|  |  |  |  |  |  |  |  |  |  |  |  |  |  |  |
| 7-9 years n=709 | 41.8 | 36.2 | 38.1 | 32 | 37.6 | 40.4 | 44.4 | 48.2 | 52.7 | 46.3 | 44.7 | 44.2 | 46.5 | 59.9 |
| n=3,382 | 36 | 30.8 | 32.6 | 26.9 | 32.1 | 34.7 | 38.4 | 41.9 | 46.1 | 40.2 | 38.6 | 38.2 | 40.4 | 52.9 |
|  | 48.3 | 42.3 | 44.3 | 37.7 | 43.8 | 46.8 | 51.1 | 55.1 | 59.9 | 53.1 | 51.4 | 50.9 | 53.4 | 67.6 |
|  | 3.1 | 2.9 | 3 | 2.7 | 3 | 3.1 | 3.2 | 3.4 | 3.5 | 3.3 | 3.2 | 3.2 | 3.3 | 3.8 |
|  |  |  |  |  |  |  |  |  |  |  |  |  |  |  |
| 9-12 years n=335 | 34.3 | 23.4 | 26.9 | 25.9 | 31.3 | 32.3 | 36.8 | 39.8 | 43.3 | 36.3 | 45.3 | 66.2 | 40.8 | 50.2 |
| n=1,875 | | | | | | | | | | | | | | |
|  | 43.2 | 30.8 | 34.8 | 33.6 | 39.8 | 40.9 | 45.9 | 49.3 | 53.1 | 45.4 | 55.3 | 78.1 | 50.4 | 60.8 |
|  | 4.1 | 3.4 | 3.7 | 3.6 | 3.9 | 4 | 4.3 | 4.4 | 4.6 | 4.3 | 4.7 | 5.7 | 4.5 | 5 |
|  |  |  |  |  |  |  |  |  |  |  |  |  |  |  |
| more than 12 years n=49 | 27.2 | 23.8 | 10.2 | 27.2 | 20.4 | 3.4 | 23.8 | 44.2 | 40.8 | 23.8 | 27.2 | 27.2 | 27.2 | 20.4 |
| n=287 | 12.8 | 10.6 | 2.8 | 12.8 | 8.5 | 0.3 | 10.6 | 24.8 | 22.3 | 10.6 | 12.8 | 12.8 | 12.8 | 8.5 |
|  | 51.3 | 46.8 | 27.2 | 51.3 | 42.1 | 15.9 | 46.8 | 73.5 | 69.1 | 46.8 | 51.4 | 51.4 | 51.3 | 42.1 |
|  | 9.6 | 9 | 5.9 | 9.6 | 8.3 | 3.4 | 9 | 12.3 | 11.8 | 9 | 9.6 | 9.6 | 9.6 | 8.3 |
|  |  |  |  |  |  |  |  |  |  |  |  |  |  |  |
| *Six- monthly time intervals from time of diagnosis (0) to death* | *7.5* | *8* | *8.5* | *9* | *9.5* | *10* | *10.5* | *11* | *11.5* | *12* | *12.5* | *13* | *13.5* | *14* |
| 7-9 years n=709 | 73.7 | 99.6 | 109.7 | 164.3 |  |  |  |  |  |  |  |  |  |  |
| n=3,382 | 65.9 | 87.7 | 92.6 | 128.9 |  |  |  |  |  |  |  |  |  |  |
|  | 82.1 | 112.7 | 129 | 206.7 |  |  |  |  |  |  |  |  |  |  |
|  | 4.1 | 6.4 | 9.3 | 19.8 |  |  |  |  |  |  |  |  |  |  |
|  |  |  |  |  |  |  |  |  |  |  |  |  |  |  |
| 9-12 years n=335 | 46.3 | 49.3 | 52.7 | 55.7 | 78.7 | 82.7 | 79.7 | 82 | 161.3 | 243.3 |  |  |  |  |
| n=1,875 | 37.6 | 40.3 | 43.4 | 46.1 | 66.3 | 67.8 | 62.1 | 59.2 | 116.9 | 124.9 |  |  |  |  |
|  | 56.4 | 59.7 | 63.5 | 66.8 | 92.8 | 99.8 | 100.7 | 110.9 | 217.3 | 431.6 |  |  |  |  |
|  | 4.8 | 5 | 5.1 | 5.3 | 6.8 | 8.1 | 9.8 | 13.1 | 25.5 | 76.9 |  |  |  |  |
|  |  |  |  |  |  |  |  |  |  |  |  |  |  |  |
| more than 12 years n=49 | 20.4 | 37.4 | 37.4 | 20.4 | 34 | 51 | 57.8 | 61.2 | 51 | 68 | 64 | 86.9 | 51.9 | 84.2 |
| n=287 | 8.5 | 19.9 | 19.9 | 8.5 | 17.5 | 29.8 | 35 | 37.6 | 29.8 | 42.9 | 38.7 | 51.7 | 23.1 | 43.2 |
|  | 42.1 | 64.8 | 64.8 | 42.1 | 60.3 | 82 | 90.5 | 94.7 | 82 | 103 | 100.1 | 137.7 | 101.8 | 149.3 |
|  |  |  |  |  |  |  |  |  |  |  |  |  |  |  |
|  | 8.3 | 11.3 | 11.3 | 8.3 | 10.8 | 13.2 | 14 | 14.4 | 13.2 | 15.2 | 15.5 | 21.7 | 19.6 | 26.6 |
|  |  |  |  |  |  |  |  |  |  |  |  |  |  |  |
| *Six- monthly time intervals from time of diagnosis (0) to death* | *14.5* | *15* | *15.5* |  |  |  |  |  |  |  |  |  |  |  |
| more than 12 years n=49 | 24.7 | 35.7 | 10.1 |  |  |  |  |  |  |  |  |  |  |  |
| n=287 | 4.9 | 9.9 | 0.9 |  |  |  |  |  |  |  |  |  |  |  |
|  | 79.1 | 95.3 | 47.1 |  |  |  |  |  |  |  |  |  |  |  |

Table 3. Incidence rates and 95% CIs for unplanned hospital admissions of people with dementia who were alive by the study end (n=6,544) – Corresponding to Manuscript, Figure 3

| Six- monthly time intervals from time of diagnosis (0) to death | 0.5 | 1 | 1.5 | 2 | 2.5 | 3 | 3.5 | 4 | 4.5 | 5 | 5.5 | 6 | 6.5 | 7 |
| --- | --- | --- | --- | --- | --- | --- | --- | --- | --- | --- | --- | --- | --- | --- |
| Duration between diagnosis and death (number of people) (number of admissions) | Rate  95% CIs Lower limit  95% CIs Upper Limit  Standard Error | | | | | | | | | | | | | |
|  |  |  |  |  |  |  |  |  |  |  |  |  |  |  |
|  |  |  |  |  |  |  |  |  |  |  |  |  |  |  |
| between 1-2 years n=1,279 | 76.5 | 65.9 | 58.1 | 67.4 |  |  |  |  |  |  |  |  |  |  |
| n=1,565 | 70.5 | 60.4 | 52.2 | 56.7 |  |  |  |  |  |  |  |  |  |  |
|  | 82.9 | 71.9 | 64.6 | 79.5 |  |  |  |  |  |  |  |  |  |  |
|  | 3.2 | 2.9 | 3.2 | 5.8 |  |  |  |  |  |  |  |  |  |  |
|  |  |  |  |  |  |  |  |  |  |  |  |  |  |  |
| 2-3 years n=712 | 63.3 | 56.9 | 54.1 | 56.7 | 58.4 | 49.3 |  |  |  |  |  |  |  |  |
| n=2,156 | 57.8 | 51.7 | 49.1 | 51.5 | 52.3 | 39.9 |  |  |  |  |  |  |  |  |
|  | 69.2 | 62.5 | 59.6 | 62.2 | 65 | 60.4 |  |  |  |  |  |  |  |  |
|  | 2.9 | 2.8 | 2.7 | 2.7 | 3.2 | 5.2 |  |  |  |  |  |  |  |  |
|  |  |  |  |  |  |  |  |  |  |  |  |  |  |  |
| 3-4 years n=607 | 52.6 | 44.9 | 50.4 | 54.6 | 52.6 | 56.7 | 55.8 | 49.3 |  |  |  |  |  |  |
| n=2,118 | 46.9 | 39.7 | 44.8 | 48.9 | 46.9 | 50.8 | 49 | 38.3 |  |  |  |  |  |  |
|  | 58.7 | 50.6 | 56.4 | 60.9 | 58.7 | 63.1 | 63.3 | 62.5 |  |  |  |  |  |  |
|  | 3 | 2.8 | 2.9 | 3.1 | 3 | 3.1 | 3.6 | 6.2 |  |  |  |  |  |  |
|  |  |  |  |  |  |  |  |  |  |  |  |  |  |  |
| 4-5 years n=486 | 56.7 | 51.3 | 47.7 | 43.7 | 53.2 | 43.9 | 52.7 | 52.5 | 47.2 | 37.8 |  |  |  |  |
| n=1,889 | 49.9 | 44.8 | 41.5 | 37.7 | 46.6 | 37.9 | 46.1 | 45.9 | 40.2 | 27.7 |  |  |  |  |
|  | 64.3 | 58.5 | 54.7 | 50.3 | 60.5 | 50.6 | 60 | 59.7 | 55.1 | 50.4 |  |  |  |  |
|  | 3.7 | 3.5 | 3.4 | 3.2 | 3.6 | 3.2 | 3.5 | 3.5 | 3.8 | 5.8 |  |  |  |  |
|  |  |  |  |  |  |  |  |  |  |  |  |  |  |  |
| 5-6 years n=413 | 53.8 | 46.7 | 43 | 49.8 | 43.8 | 46.1 | 39.2 | 47.8 | 42.1 | 46.4 | 47.7 | 28.5 |  |  |
| n=1,748 | 46.5 | 39.9 | 36.5 | 42.8 | 37.3 | 39.4 | 33.1 | 41 | 35.7 | 39.7 | 39.8 | 18.7 |  |  |
|  | 62 | 54.3 | 50.3 | 57.7 | 51.2 | 53.6 | 46.2 | 55.5 | 49.3 | 54 | 56.7 | 41.6 |  |  |
|  | 3.9 | 3.7 | 3.5 | 3.8 | 3.5 | 3.6 | 3.4 | 3.7 | 3.5 | 3.6 | 4.3 | 5.8 |  |  |
|  |  |  |  |  |  |  |  |  |  |  |  |  |  |  |
| 6-7 years n=330 | 39.8 | 32.5 | 30.7 | 33.9 | 21.3 | 28.3 | 31.8 | 38.4 | 39.1 | 38.1 | 41.6 | 38.4 | 39.7 | 48.9 |
| n=1,301 | 33 | 26.4 | 24.8 | 27.6 | 16.5 | 22.6 | 25.8 | 31.7 | 32.4 | 31.4 | 34.6 | 31.7 | 31.7 | 34.6 |
|  | 47.7 | 39.6 | 37.7 | 41.2 | 27.2 | 35 | 38.8 | 46.1 | 46.9 | 45.8 | 49.6 | 46.1 | 49 | 67.2 |
|  | 3.7 | 3.4 | 3.3 | 3.4 | 2.7 | 3.1 | 3.3 | 3.7 | 3.7 | 3.6 | 3.8 | 3.7 | 4.4 | 8.3 |
|  |  |  |  |  |  |  |  |  |  |  |  |  |  |  |
| 7-9 years n=379 | 44.4 | 29.9 | 33 | 36 | 35.1 | 41 | 33.9 | 32.7 | 29.6 | 35.1 | 40 | 33 | 32 | 34.5 |
| n=1,801 |  |  |  |  |  |  |  |  |  |  |  |  |  |  |
|  | 37.6 | 24.4 | 27.2 | 29.9 | 29.1 | 34.4 | 28 | 26.9 | 24.1 | 29.1 | 33.6 | 27.2 | 26.3 | 28.5 |
|  | 52.1 | 36.3 | 39.7 | 43 | 42 | 48.4 | 40.7 | 39.3 | 35.9 | 42 | 47.4 | 39.7 | 38.7 | 41.3 |
|  | 3.7 | 3 | 3.2 | 3.3 | 3.3 | 3.6 | 3.2 | 3.2 | 3 | 3.3 | 3.5 | 3.2 | 3.1 | 3.3 |
|  |  |  |  |  |  |  |  |  |  |  |  |  |  |  |
| 9-12 years n=387 | 30.9 | 32.7 | 29.2 | 23.9 | 28.3 | 21.5 | 23.3 | 31.8 | 26.8 | 25.6 | 26.2 | 27.4 | 27.1 | 30.9 |
| n=1,991 | 25.4 | 27 | 23.8 | 19.1 | 23 | 17 | 18.5 | 26.2 | 21.7 | 20.7 | 21.2 | 22.2 | 22 | 25.4 |
|  | 37.3 | 39.2 | 35.3 | 29.5 | 34.4 | 26.9 | 28.8 | 38.2 | 32.7 | 31.4 | 32.1 | 33.4 | 33.1 | 37.3 |
|  | 3 | 3.1 | 2.9 | 2.7 | 2.9 | 2.5 | 2.6 | 3.1 | 2.8 | 2.7 | 2.8 | 2.8 | 2.8 | 3 |
|  |  |  |  |  |  |  |  |  |  |  |  |  |  |  |
| more than 12 years n=99 | 23.1 | 8.3 | 11.1 | 12 | 13.9 | 11.1 | 10.2 | 13 | 11.1 | 7.4 | 13 | 4.6 | 9.3 | 14.8 |
| n=378 | 15.3 | 4.1 | 6.1 | 6.7 | 8.1 | 6.1 | 5.4 | 7.4 | 6.1 | 3.5 | 7.4 | 1.8 | 4.8 | 8.8 |
|  | 33.6 | 15.2 | 18.8 | 20 | 22.3 | 18.8 | 17.6 | 21.2 | 18.8 | 14 | 21.2 | 10.1 | 16.4 | 23.5 |
|  | 4.6 | 2.8 | 3.2 | 3.3 | 3.6 | 3.2 | 3.1 | 3.5 | 3.2 | 2.6 | 3.5 | 2.1 | 2.9 | 3.7 |
|  |  |  |  |  |  |  |  |  |  |  |  |  |  |  |
| *Six- monthly time intervals from time of diagnosis (0) to death* | *7.5* | *8* | *8.5* | *9* | *9.5* | *10* | *10.15* | *11* | *11.5* | *12* | *12.5* | *13* | *13.5* | *14* |
| 7-9 years n=379 | 35.2 | 36.7 | 42 | 34.4 |  |  |  |  |  |  |  |  |  |  |
| n=1,801 | 0 | 0 | 0 | 0 |  |  |  |  |  |  |  |  |  |  |
|  | 28.8 | 28.4 | 30.6 | 17 |  |  |  |  |  |  |  |  |  |  |
|  | 42.7 | 46.7 | 56.4 | 62.8 |  |  |  |  |  |  |  |  |  |  |
|  | 3.6 | 4.7 | 6.6 | 11.5 |  |  |  |  |  |  |  |  |  |  |
|  | 0 | 0 | 0 | 0 |  |  |  |  |  |  |  |  |  |  |
| 9-12 years n=387 | 29.2 | 28.6 | 30.6 | 30.9 | 30.9 | 25.6 | 27.2 | 24 | 32.5 | 27.6 |  |  |  |  |
| n=1,991 | 23.8 | 23.3 | 25.2 | 25.4 | 25.2 | 19.9 | 20.5 | 17.1 | 21.5 | 10.5 |  |  |  |  |
|  | 35.3 | 34.7 | 36.9 | 37.3 | 37.6 | 32.4 | 35.3 | 32.8 | 47.2 | 60.4 |  |  |  |  |
|  | 2.9 | 2.9 | 3 | 3 | 3.2 | 3.2 | 3.8 | 4 | 6.5 | 12.3 |  |  |  |  |
|  |  |  |  |  |  |  |  |  |  |  |  |  |  |  |
| more than 12 years n=99 | 14.8 | 18.5 | 17.6 | 13.9 | 11.1 | 8.3 | 16.7 | 8.3 | 9.3 | 25.3 | 13.4 | 21 | 12.7 | 18.8 |
| n=378 | 8.8 | 11.7 | 10.9 | 8.1 | 6.1 | 4.1 | 10.2 | 4.1 | 4.8 | 14.8 | 7.5 | 12.5 | 5.7 | 8.9 |
|  | 23.5 | 28 | 26.9 | 22.3 | 18.8 | 15.2 | 25.8 | 15.2 | 16.4 | 40.6 | 22.2 | 33.3 | 25 | 35.5 |
|  | 3.7 | 4.1 | 4 | 3.6 | 3.2 | 2.8 | 3.9 | 2.8 | 2.9 | 6.5 | 3.7 | 5.2 | 4.8 | 6.7 |
|  |  |  |  |  |  |  |  |  |  |  |  |  |  |  |
| *Six- monthly time intervals from time of diagnosis (0) to death* | *14.5* | *15* | *15.5* |  |  |  |  |  |  |  |  |  |  |  |
| more than 12 years n=99 | 18.4 | 6.3 | 16.5 |  |  |  |  |  |  |  |  |  |  |  |
| n=378 | 1.3 | 5.5 | 0.4 |  |  |  |  |  |  |  |  |  |  |  |
|  | 20.3 | 39.3 | 20.5 |  |  |  |  |  |  |  |  |  |  |  |
|  | 4.5 | 8.3 | 4.4 |  |  |  |  |  |  |  |  |  |  |  |

Figure 1. Unplanned hospital admission rates per person-month for six-monthly intervals of 19,221 of people with dementia. Each point indicates the rate of unplanned hospital admissions for the six-month long time interval in person-months (rates range from 0.010 – 0.334).
